# Exploring potential serum levels of Homocysteine, interleukin‐1 beta, and apolipoprotein B 48 as new biomarkers for patients with ischemic stroke

**DOI:** 10.1002/jcla.23996

**Published:** 2021-09-07

**Authors:** Behrouz Shademan, Alireza Nourazarian, Delara Laghousi, Vahidreza Karamad, Masoud Nikanfar

**Affiliations:** ^1^ Department of Medical Biology Faculty of Medicine EGE University Izmir Turkey; ^2^ Neurosciences Research Center (NSRC) Tabriz University of Medical Sciences Tabriz Iran; ^3^ Department of Biochemistry and Clinical Laboratories Faculty of Medicine Tabriz University of Medical Sciences Tabriz Iran; ^4^ Social Determinants of Health Research Center Tabriz University of Medical Sciences Tabriz Iran; ^5^ Department of Neurology Faculty of Medicine Tabriz University of Medical Sciences Tabriz Iran

**Keywords:** apolipoprotein B 48, Body Mass Index, Homocysteine, interleukin‐1β, ischemic Stroke

## Abstract

**Background:**

Stroke is the second leading cause of death worldwide with heterogeneous characteristics. The subtypes of stroke are due to different pathophysiological regulations and causes. This study aimed to investigate the correlation of serum levels of apolipoprotein B 48, interleukin‐1β and Homocysteine with BMI in patients with ischemic stroke (IS).

**Methods:**

Over one hundred controls (120) and an equal number of IS patients, including 31 women and 89 men, were recruited to participate in the case‐control study conducted at Imam Reza Hospital (Tabriz, Iran) from February 2019 to March 2020. We measured serum levels of apolipoprotein B 48, interleukin‐1β, and Homocysteine. Receiver operating characteristic analysis (ROC) was performed to evaluate the diagnostic value of these indices in patients and control groups.

**Results:**

The mean serum levels of apolipoprotein B 48, interleukin‐1β, and Homocysteine, were significantly increased in the experimental group compared to the control group with a *p*‐value of 0.001. The ROC curve analysis showed that the area under the curve for apo B48, IL −1β, hs‐CRP, and Homocysteine serum levels were 0.94, 0.98, 0.99, and 1, respectively.

**Conclusions:**

The results of our current study show that the determination of serum levels of apolipoprotein B 48, interleukin‐1β, and Homocysteine can potentially be used to monitor and diagnose IS patients. However, there was no statistically significant correlation between serum levels of apolipoprotein B 48, interleukin 1β and Homocysteine and BMI in the patient group. However, there was a statistically significant inverse correlation between serum levels of high‐sensitivity C‐reactive protein (hs‐CRP) and BMI in the patient group.

## Graphical abstract

**Background**Stroke is ranked as the second most prevalent cause of mortality worldwide with heterogeneous characteristics. The subtypes of stroke result from distinct pathophysiological regulations and reasons. The objective of this study was to evaluate the correlation of serum levels of apolipoprotein B 48, interleukin‐1β, and homocysteine with BMI in ischemic stroke (IS) patients.**Methods**Over one hundred controls (120) and an equal number of IS patients, including 31 women and 89 men, were recruited to participate in the case‐control investigation conducted at Imam Reza Hospital (Tabriz, Iran) from February 2019 to March 2020. We measured serum levels of apolipoprotein B 48, interleukin‐1β, and homocysteine. The analysis of receiver operating characteristics (ROC) was performed to evaluate the incremental diagnostic value of these indices in patients and control groups.**Results**Mean serum levels of apolipoprotein B 48, interleukin‐1β, and homocysteine were remarkably elevated in the experimental group compared with the control group with a p‐value of 0.001. The analysis of ROC unraveled the diagnostic value of apolipoprotein B‐48, interleukin‐1β, and homocysteine for IS demonstrated by a region under the ROC curve of less than 0.98, a p‐value of less than 0.001, and significant specificity and sensitivity.**Conclusions**Findings from our current study revealed that assessment of serum levels of apolipoprotein B 48, interleukin‐1β, and homocysteine could be potentially utilized to monitor and diagnose IS patients. However, there was no statistically significant correlation between serum levels of apolipoprotein B 48, interleukin 1β, and homocysteine and BMI in the patient group. However, there was a statistically significant inverse correlation between serum levels of high‐sensitivity C‐reactive protein (hs‐CRP) and BMI in the patient group.

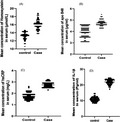

## 1 INTRODUCTION

Stroke is the second leading cause of death in industrialized countries. It is estimated that nearly 15 million people worldwide fall victim to a stroke, of whom 5 million die and the remainder suffer from permanent disability.^1^, ^2^ Stroke is a multifaceted disease with different subtypes that have various physiopathologic etiologies and mechanisms. A moderate decline in the total number of deaths from strokes has been observed in industrialized countries. Nevertheless, stroke remains the leading cause of death and mortality worldwide.^3^

IS is a condition in which the blood supply to the brain fails due to the formation of blood clots in the vessels and lack of nutrient and oxygen supply, resulting in brain tissue impairment.^3^ However, appropriate preventive/therapeutic strategies include lifestyle modification, dietary guidelines, and pharmaceutical interventions. The prevalence of stroke remains high, especially in developed countries with a significant financial burden.^4^ It should be noted that predicting the outcome of IS is of paramount importance to clinicians, researchers, and patients. Previous clinical prognostic approaches have failed to accurately predict the outcome of IS in patients. In this context, blood markers associated with pathophysiological aspects of IS, including inflammation, glial and neuronal damage, cardiovascular disease, and hemostasis, could be considered to improve the utility of clinical prognostic approaches.^5^ Diabetes mellitus and obesity are potential risk factors for cardiovascular disease.

High blood triglyceride levels, also known as hypertriglyceridemia (HTG), are a typical dyslipidemia. HTG favors the development and promotion of atherosclerosis, which eventually leads to CVD. Therefore, HTG is a potential predisposing factor for IS.^6^ In this context, numerous studies have attempted to uncover the correlation between IS and HTG. Numerous studies have shown that triglyceride levels after eating (without fasting) may be a more reliable biomarker for IS and atherosclerosis disease than triglyceride levels during fasting.^7^

A variety of factors contribute to the increase in triglyceride levels after a meal. However, normalization/standardization has not been considered in the measurement of triglyceride concentration after a meal. An alternative screening for atherosclerotic disease and IS may be apolipoprotein B 48 (apo B48). Enterocytes also called absorptive cells that line the small intestine, produce apo B48, an element necessary for forming chylomicrons. In each chylomicron particle, there is one Apo B48.^6^

In addition, some studies have shown that fasting apo‐B 48 level can be used as a surrogate marker for measuring lipidemia in the non‐fasting state. Therefore, the fasting apo‐B 48 level could be a potential biological marker for atherosclerotic diseases such as CVD and IS.^6^, ^7^, ^8^ Homocysteine is considered a causative factor in atherogenesis and thrombus formation by causing endothelial impairment, vascular smooth muscle proliferation, and coagulopathy. Abnormally elevated blood levels of Homocysteine, also known as hyperhomocysteinemia (HH), have been associated with CVD and cerebrovascular disorders.^9^ It has been reported that HH is an independent predisposing factor for IS in adolescent Asians.^10^

One of the essential proinflammatory cytokines is interleukin‐1 (IL −1), which plays an important role during the inflammatory process. IL −1α and IL −1β belong to the IL −1 superfamily.^11^ Numerous studies have shown that IL −1β is a neurotoxic element in IS models and accelerates patients with acute IS.^12^ Some studies have found that IL −1 is a critical chemical compound involved in hypoxia, ischemia, stress responses, and infection. IL −1β leads to inflammatory responses in the brain by stimulating cells to produce neurotoxic elements, resulting in necrosis of brain tissue.^11^, ^12^

High‐sensitivity C‐reactive protein (hs‐CRP) is a sensitive marker of arterial wall inflammation and tissue damage.^13^, ^14^ CRP is a glycoprotein produced by the liver that plays a critical role in developing atherosclerotic disease in the heart and cerebral circulation. In addition, elevated hsCRP levels have been associated with acute stroke as a sign of infection and inflammation.^15^, ^16^

Although body mass index (BMI) is a known risk factor for vascular disease, the impact of BMI on stroke has been debated. Several studies have shown that obesity is associated with better functional outcomes after stroke. It is unknown whether obesity plays a role in stroke patients.^17^ The present study examined the correlation between serum concentrations of apo B48, IL −1β, and Homocysteine with BMI in IS patients.

## 2 MATERIALS AND METHODS

### 2.1 Study population

This study is a retrospective case‐control study. We selected 155 prototypes. We excluded samples from patients with the previous concurrent IS, neurologic disease (epilepsy), infarcts, hypothyroidism, circulatory disorders, pregnancy, cerebral sinus thrombosis, postnatal condition, ischemic CVD, brain tumors, renal disease, use of oral contraceptives, use of drugs affecting serum concentrations of folic acid, B12, and Homocysteine, deficiency of vitamins including folic acid and B12, and history of migraine. The control group was selected from patients whose presence of IS could be excluded by a neurologist based on MRI and CT results. The inclusion criteria were primary diagnosis of acute IS, completion of questionnaires, and consent to participate in the study. The control group was selected from patients in whom IS was excluded by a neurologist based on MRI and CT results.

The control group, consisting of 120 subjects (31 women and 89 men) without stroke and neurological diseases and degeneration of the nervous system, and the experimental group, consisting of 120 subjects (31 women and 89 men) affected by IS, were studied in a case‐control study at Imam Reza Hospital, Tabriz, Iran, during 2019 (February)‐2020 (March). Investigators registered patients at baseline IS; magnetic resonance imaging (MRI) and computed tomography (CT) were used for radiological diagnosis IS. Neurologists confirmed the disease. Infarct size was assessed with a threshold of 2.0 cm (an infarct larger than 2.0 cm was considered IS).^18^ Ninety‐five patients had IS right cerebral hemisphere disease, and 25 patients suffered from left cerebral hemisphere disease IS.

### 2.2 Ethics statement

The study described here was approved by the Ethics Committee of Tabriz University of Medical Sciences (IR. TBZMED. REC.1398.483), and patients also provided written informed consent.

### 2.3 Blood sample collection

The sampling place was the laboratory of Imam Reza Hospital in Tabriz, and sampling was performed from 2019 (February) to 2020 (March). After fasting for 12 hours, 10 ml of venous blood was collected by venipuncture (by an expert in laboratory science) and centrifuged at 1200 *g* after a clotting time of 1–2 h at room temperature. The serum obtained from the blood samples was immediately poured into EP tubes and stored at 70°C for analysis.

### 2.4 Biochemical experiments in serum samples

Serum concentrations of folate and vitamin B12 were quantified using MyBioSource Elisa kits (MBS700741 and MBS169369, USA, respectively). The detection limits for folate and vitamin B12 ELISA kits were 1 µmol/L and 1 pg/mL, respectively. The Pars Azmoon triglyceride kits (GPO‐PAP, cat. no. 132 504 H917), cholesterol kits (cat. no. 110 500 BT), and HDL kits (cat. no. 1050012) were also used to estimate lipid profiles in the Hitachi 917 (an automated biochemistry analyzer). The Friedewald formula was used to calculate low‐density lipoproteins (LDL).^19^, ^20^

### 2.5 Measuring serum concentrations of Homocysteine, IL‐1β, Apo B48, and hs‐CRP

The kits were purchased from Elabscience Biotechnology Inc (Cat. No. E‐BC‐K143, China), (Cat. No. E‐ EL ‐H0149, China), MyBioSource (Cat. No. MBS 037744, USA), and MyBioSource Elisa kits (Cat. No. MBS3800421, USA) used to measure Homocysteine, IL −1β, Apo B48, and hs‐CRP, with a detection limit of 10 µmol/L,4.69 pg/ml, 1.0 µg/ml, and 0.1 mg/L, respectively.

### 2.6 BMI measurements

BMI was determined by measuring the height and weight of patients, and a BMI greater than/equal to 30 kg/m^2^ was considered obesity.^21^

### 2.7 Statistical analysis

Graph Pad Prism 8 was used to analyze the data. The Kolmogorov–Smirnov test was also used to assess the normality of the (quantitative) variables. Mean ± SD and percentage frequency were used to indicate quantitative and qualitative variables, respectively. In addition, logistic regression and Mann–Whitney U examination were used for data analysis. ROC curves served the purpose of identifying the most appropriate thresholds (stopping values) to distinguish IS patients from healthy individuals. Spearman test was performed to evaluate the correlation of serum concentrations of Apo B48, IL −1β and Homocysteine with BMI in both groups (patients or healthy subjects). In the study groups, a P‐value of less than 0.05 was considered to indicate a significant difference.

## 3 RESULTS

### 3.1 Demographic data of the subject

The study included 120 samples from each group (120 from patients and 120 from healthy subjects). As shown in Table 1, there were no statistically significant differences between the groups with respect to age, smoking, diabetes, and sex. However, there were significant differences between groups for BMI and hypertension, with *p*‐values <0.001.

**TABLE 1:** Demographic data of the patients with ischemic stroke and healthy individuals.

Variable	Cases(120)	Control(120)	*p*‐value
Gender, n (%)			
Male	85(70.1%)	83(69.1%)	1
Female	35 (29.1%)	37 (30.8%)	
Mean age (SD)	58.2 ± 8.5	55.1 ± 6.6	1
Cigarette, n (%)	12(10%)	10(8.3%)	>0.05
Diabetic, n (%)	15(12.5%)	11(9.1%)	>0.05
Hypertensives (%)	56(46.6%)	22(18.3%)	<0.001*
BMI	23.3 ± 1.2	21.4 ± 3.5	<0.001*

Abbreviation: BMI, Body mass index.

* P‐value was reported based on the Mann–Whitney *U* test.

### 3.2 Assessing the concentrations of biochemical factors

As Table 2 shows, there were remarkable differences in serum concentrations of HDL‐C, chol, folic acid, vitamin B12, apo B48, and IL −1β in IS patients and healthy subjects. However, there were no raemarkable differences in the serum concentrations of TG and LDL in both groups.

**TABLE 2:** Comparison of the blood level of the study biomarkers between control groups and ischemic strokes patients

	Control groups			patients with ischemic stroke			*p*‐value*
Variable	Med (P_25_ to P_75_)	Mean (SD)	Mean Rank	Med (P_25_ to P_75_)	Mean (SD)	Mean Rank	
Homocysteine (μmol/L)	13.4 (12.6–13.6)	13.2(0.82)	61.15	16.3 (15.3–16.8)	16.1(1.20)	175.85	<0.0001
IL‑1β(Pg/ml)	10.6 (9.6–11.6)	10.8(1.42)	59.50	22.2 (20.7–23.1)	21.8 (1.56)	177.50	<0.0001
Apo B48(μg/ml)	4.1 (3.5–4.3)	3.9 (0.49)	66.53	4.7 (4.6–4.9)	4.7 (0.25)	170.47	<0.0001
hs‐CRP(mg/L)	1.7 (1.5–1.9)	1.7(0.6)	59.94	2.7 (2.6–2.9)	2.7 (0.21)	177.06	<0.0001
Vit B12(Pg/ml)	440 (395–460)	428.5 (37.0)	177.50	280 (260–290)	278.2(22.9)	59.50	<0.0001
Folic acid (ng/ml)	16.9 (16.3–18.3)	16.9 (1.16)	177.38	10.5 (9.7–11.3)	10.5(1.14)	59.62	<0.0001
LDL‐C(mg/dl)	87.1 (84.3–87.9)	86.2 (1.94)	118.35	87.3 (84.3–88)	86.2(1.99)	118.65	0.973
TG(mg/dl)	96 (90–120)	100.8(17.7)	114.31	98 (90–120)	104.1(19.4)	122.69	0.343
CHOL(mg/dl)	136 (111–140)	126.1(21.0)	100.92	138 (132–142.7)	137.4(8.38)	136.08	<0.0001
HDL‐C(mg/dl)	37 (35–42)	37.9(3.42)	173.49	29 (25.5–32)	28.7(4.32)	63.51	<0.0001

Abbreviations: IL‑1β, Interleukin 1 beta; Apo B48, Apolipoprotein B 48; hs‐CRP, High Sensitivity C‐Reactive Protein; LDL‐C, low‐density lipoprotein cholesterol; TG, triglyceride; CHOL, Cholesterol; HDL‐C, High ‐density lipoprotein cholesterol.

* Mann–Whitney *U* test was used.

### 3.3 Assessing the serum concentrations of Homocysteine

**FIGURE 1:**
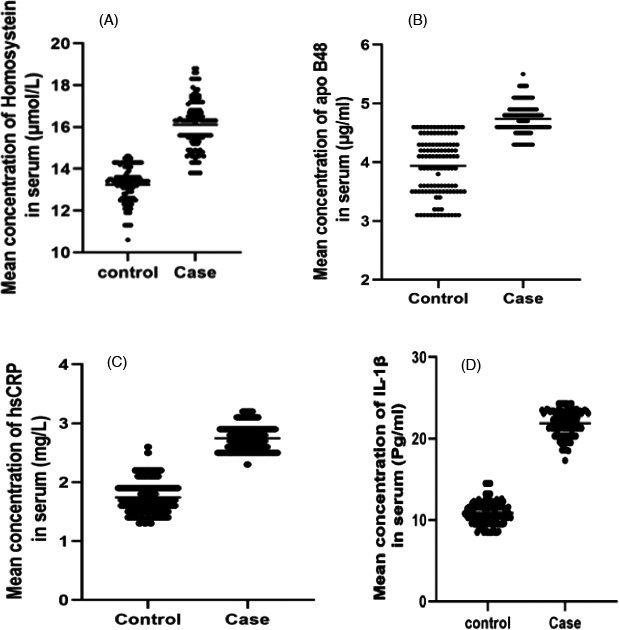
The serum concentration of Homocysteine (A), Apo B48 (B), hs‐CRP (C), and IL 1β (D) among Patients with ischemic stroke and control groups. The Mann–Whitney U test did the statistical analysis. IL‑1β, Interleukin 1 beta; Apo B48, Apolipoprotein B‐48; hsCRP: High Sensitivity C‐Reactive Protein

As Figure 1 shows, serum Homocysteine level was significantly increased in IS patients (16.1 ± 1.20 μmol/L) compared to that of the healthy group (13.2 ± 0.8 μmol/L) with a *p*‐value <0.001.

### 3.4 Assessing the serum concentrations of apo B48

As Figure 1 shows, serum concentrations of apo B48 were significantly increased in IS patients (4.7 ± 0.25 μg/ml) compared to those in the healthy subjects group (3.9 ± 0.49 μg/ml) with a P‐value of 0.0001.

### 3.5 Assessing the serum concentrations of hs‐CRP

As shown in Figure 1, serum concentrations of hs‐CRP were significantly elevated (2.7 ± 0.21 mg/L) in the IS patients compared to the healthy group (1.7 ± 0.6 mg/L) with a *p*‐value of 0.0001.

### 3.6 Assessing the serum concentrations of IL‐1β

As shown in Figure 1, serum concentrations of IL −1β were significantly elevated (21.8 ± 1.5 Pg/ml) in the IS patients compared to the healthy group with a *p*‐value of 0.0001.

### 3.7 Analyzing the ROC curve

**FIGURE 2:**
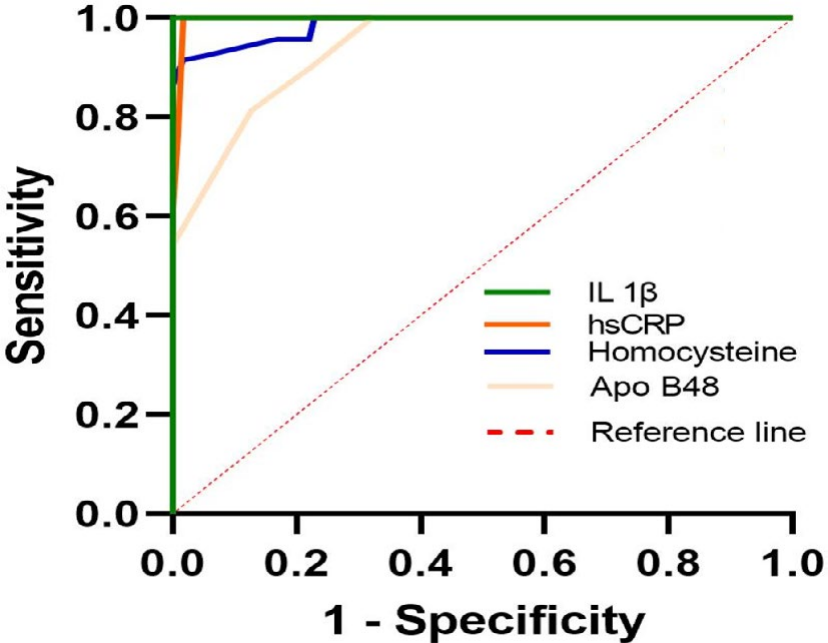
ROC curve analysis for the combination of serum concentrations of Homocysteine, Apo B48, hsCRP, IL 1β in patients with Ischemic stroke. IL‑1β, Interleukin 1 beta; Apo B48, Apolipoprotein B‐48; hsCRP, High Sensitivity C‐Reactive Protein

A ROC curve analysis was performed to investigate the diagnostic potential of serum elements in IS patients. According to Table 3, which shows the results of ROC curve analysis, the AUC values for apo B48, IL −1β, hs‐CRP, and Homocysteine were 0.94, 0.98, 0.99, and 1 of 95%, respectively. It is noteworthy that the results are statistically significant with *p*‐values less than 0.05, considering that the areas under the curve of the results (considered as 95% confidence intervals) do not have an AUC value of 0.5, showing that all biological markers are perfectly suitable to be used as identifiers of patients with IS as well as healthy individuals (Figure 2). Table 4 shows the cut‐off value of IL −1β, hs‐CRP, apo B48, and Homocysteine according to the Youden index results.

**TABLE 3:** The area under the Curve values of ROC curve analysis for Homocysteine, Apo B48, hs‐CRP, IL 1β in patients with Ischemic stroke

Variable(s)	Area	Std. Error	95% CI	*p*‐Value*
IL‑1β(Pg/ml)	1.000	0.000	1.000 to 1.000	<0.0001
hs‐CRP(mg/L)	0.9963	0.002989	0.9904 to 1.000	<0.0001
Homocysteine(μmol/L)	0.9860	0.005255	0.9757 to 0.9963	<0.0001
Apo B48(μg/ml)	0.9405	0.01339	0.9142 to 0.9667	<0.0001

Abbreviations: IL‑1β, Interleukin 1 beta; Apo B48, Apolipoprotein B 48; hs‐CRP, High Sensitivity C‐Reactive Protein.

* Null hypothesis: true area =0.5.

**TABLE 4:** The sensitivity and specificity of the Homocysteine, apo B48, hs‐CRP, IL 1β in the detection of stroke according to the Youden Index

	Cut‐off point value	Sensitivity	Specificity
IL‑1β(Pg/ml)	≥15.9	1	1
hs‐CRP(mg/L)	≥2.25	1	0.9831
Homocysteine(μmol/L)	≥14.55	0.915	0.9831
apo B48(μg/ml)	≥4.55	0.814	0.8729

Abbreviations: IL‑1β, Interleukin 1 beta; apo B48, apolipoprotein B 48; hs‐CRP, High Sensitivity C‐Reactive Protein.

### 3.8 Spearman's correlation

The results of the Spearman correlation test showed that there were only small and statistically non‐significant correlations between serum levels of apo‐B48, IL −1β and Homocysteine and BMI in IS patients. However, there was a small but statistically significant inverse correlation between BMI and serum levels of hs‐CRP, according to Table 5.

**TABLE 5:** The correlation between serum concentration of Homocysteine, IL‐1β, apo B48, hs‐CRP and BMI among patients with Ischemic stroke

Variable	*N*	Correlation coefficient	*p*‐value^*^
Homocysteine (μmol/L)	120	−0.067	0.474
IL‑1β(Pg/ml)	120	0.036	0.695
apo B 48(μg/ml)	120	0.035	0.703
hs‐CRP(mg/L)	120	−0.198	0.032

Spearman test was used.

## 4 DISCUSSION

The current study examined the correlation between serum concentrations of apo B48, IL −1β, and Homocysteine with BMI in IS patients. Previously, it was found that there was a correlation between a slight increase in plasma Homocysteine and IS. Several studies have shown that Homocysteine can increase the risk of IS two‐ to threefold.^22^, ^23^, ^24^ Compared to the entire group, serum concentration of Homocysteine increased in IS patients during the study. During the study, patients did not receive vitamin supplementation that could affect/reduce homocysteinemia. However, it is not clear whether dietary changes affect homocysteinemia or not.^25^, ^26^ In the first 27 h after IS, hyperhomocysteinemia was shown to have a negative predictive value for practical outcomes at 18, 12, and 6 months, based on the report of scores obtained from the BI mRS and NIHSS. The Multivariate and univariate analysis showed that serum concentration of Homocysteine was associated with serum level of vitamin B12, indicating a significant association between Homocysteine and B12.^27^ Diets limited to vegetables and lacking dairy products are usually deficient in vitamin B12; in addition, autoimmune elements and genetic factors may play a crucial role in vitamin B12 levels.^28^ Hyperhomocysteinemia has been reported to contribute to significant vascular disease and severe stroke.

However, a review of the United Kingdom population found that Homocysteine is generally correlated with low vascular complications.^9^, ^27^ Serum Homocysteine levels have been reported to increase with age.^28^ However, no association between Homocysteine and age was found in the present study. The patients who participated in this study were predominantly over 50 years of age. Thus, from a statistical point of view, aging did not affect the results of this study.

Elevated hsCRP levels (>3.0 mg/L) were found in 32% of the study group. In addition, elevated hs‐CRP levels independently predicted recurrent stroke during a one‐year follow‐up period. In a randomized trial, there was no association between hsCRP levels and antiplatelet drugs.^29^ Our results showed that serum hs‐CRP levels increased significantly in IS patients compared with the healthy group. Consequently, this biomarker can be used to monitor the therapeutic process.

In the current study, we found that fasting levels of Apo B 48 were elevated in IS patients. Other studies have also found cytokine secretion increases during brain injury due to the accelerated production of cytokines by neurons, glia, and inflammatory cells.^30^ This study shows that fasting levels of apo B48 are correlated with IS, suggesting that lipoproteins containing apo B48 are involved in the development of atherosclerosis.^31^

In addition, numerous studies have described plausible mechanisms. apo B48 contains a binding residue to proteoglycans of the arterial wall.^31^ Consequently, apo B48 has been observed in plaques obtained from carotid and femoral endarterectomy specimens formed during atherosclerosis.^32^ In vitro studies have also shown that chylomicron remnants are generally taken up by murine peritoneal macrophages and human monocyte‐derived macrophages via different mechanisms.^33^ Suggesting that fasting apo B48 may be a novel biomarker for IS and a new therapeutic intervention target. In Western countries with a very high BMI, the reduced risk of developing IS was not observed. The increased risk of IS or hemorrhagic stroke in the male population with BMI above the standard range (22 to 23 kg/m^2^) was rejected after adjustment for blood pressure, glucose, and cholesterol, suggesting that BMI may have a significant impact on the subtypes of stroke in the population.^34^ Other studies have also shown a correlation between different subtypes of stroke and BMI.^35^, ^36^

However, numerous studies on the obesity paradox have shown that obesity and being overweight can be risk factors for vascular complications. This study showed that the serum concentration of IL −1β was strikingly increased in IS patients compared with healthy individuals. Reportedly, the expression level of IL −1β is high in cerebral infarction patients, characterized by various neurological deficits compared to mild cerebral infarction patients.^30^ Other studies have also found that after brain injury, cytokine levels are increased due to increased cytokine production by neurons, glia, and inflammatory cells.^37^, ^38^ Among these cytokines, IL −1β correlated with the worsening of impairment caused by stroke.^39^

In contrast, other studies have reported that a slight increase in IL −1β may be a helpful factor in protecting the brain from ischemia by activating astrocytes that maintain antioxidant defenses in the brain during ischemic reperfusion injury and by protecting against ROS damage.^40^ Therefore, it can be postulated that cytokines involved in the inflammatory response, such as IL −1 β in conjunction with other predisposing factors for stroke, could negatively or positively influence stroke development, severity, and pathogenesis. The notable predisposing factors are obesity, dyslipidemia, diabetes mellitus, and aging.

There are some limitations in the current study. However, the study disregarded factors that may have affected the quantification of cytokines, such as medications, circadian rhythm, environmental factors, and stress. First, no infectious diseases were detected in the patients during the study. Second, most patients who participated in this study experienced Imam Reza Hospital from Tabriz University Medical Sciences for the first time. However, some patients were already receiving medical therapeutics before their hospitalization, and it was not clear whether they were receiving steroid medications or not. This could lead to a bias in the results for inflammatory cytokines. Third, blood samples were taken from the patients at admission.

## 5 CONCLUSION

Overall, serum levels of hs‐CRP, apo B48, IL −1β, and Homocysteine were significantly higher in IS patients than in the control group. Ultimately, the ROC curve results indicated that the serum levels of hs‐CRP, apo B48, IL −1β, and Homocysteine in IS patients could be used as novel biomarkers to predict or therapeutically guide IS patients along with other biomarkers. In addition, a negative correlation was found between serum levels of BMI and hs‐CRP in the current study. Because the sample size in this study was not large enough, upcoming studies should conduct a large‐scale investigation to reveal the correlation of serum levels of apo‐B48, IL −1β, and Homocysteine with BMI in IS patients.

## CONFLICTS OF INTEREST

There are no conflicts of interest.

## AUTHOR CONTRIBUTIONS

AN and MN contributed to the study design; DL contributed to the analysis and interpretation of the data; VK, BS, and AN contributed to revising the manuscript content; and AN approved the final version of the manuscript.

## Data Availability

All data obtained in the study can be accessed upon request.
